# The Potential Use of THP-1, a Monocytic Leukemia Cell Line, to Predict Immune-Suppressive Potency of Human Bone-Marrow Stromal Cells (BMSCs) In Vitro: A Pilot Study

**DOI:** 10.3390/ijms241713258

**Published:** 2023-08-26

**Authors:** Jiaqiang Ren, Gergely Szombath, Lynn Vitale-Cross, David F. Stroncek, Pamela G. Robey, Anna Hajdara, Ildiko Szalayova, Balazs Mayer, Daniel Martin, Eva Mezey, Krisztian Nemeth

**Affiliations:** 1 Center for Cellular Engineering, National Institutes of Health, Bethesda, MD 20892, USA; jiaqiang.ren@nih.gov (J.R.); dstroncek@cc.nih.gov (D.F.S.); 2 Department of Internal Medicine and Hematology, Semmelweis University, 1085 Budapest, Hungary; szombath.gergely@med.semmelweis-univ.hu; 3 Károly Rácz Doctoral School of Clinical Medicine, Semmelweis University, 1085 Budapest, Hungary; 4 Adult Stem Cell Section, National Institutes of Dental and Craniofacial Research, National Institutes of Health, Bethesda, MD 20892, USA; lcross@dir.nidcr.nih.gov (L.V.-C.); ildiko.szalayova@nih.gov (I.S.); 5 Skeletal Biology Section, National Institute of Dental and Craniofacial Research, National Institutes of Health, Bethesda, MD 20892, USA; probey@dir.nidcr.nih.gov; 6 Roska Tamás Doctoral School of Sciences and Technology, Faculty of Information Technology and Bionics, Pázmány Péter Catholic University, 1083 Budapest, Hungary; anna.hajdara@itk.ppke.hu; 7 Department of Dermatology, Venereology and Dermatooncology, Semmelweis University, 1085 Budapest, Hungary; mayer.balazs@med.semmelweis-univ.hu; 8 Genomics and Computational Biology Core, NIDCR, National Institutes of Health, Bethesda, MD 20892, USA; daniel.martin@nih.gov

**Keywords:** human bone marrow, MSC immune suppression, MLR assay, T-cell proliferation, IL-10, macrophage polarization

## Abstract

Adoptive transfer of cultured BMSCs was shown to be immune-suppressive in various inflammatory settings. Many factors play a role in the process, but no master regulator of BMSC-driven immunomodulation was identified. Consequently, an assay that might predict BMSC product efficacy is still unavailable. Below, we show that BMSC donor variability can be monitored by IL-10 production of monocytes/macrophages using THP-1 cells (immortalized monocytic leukemia cells) co-cultured with BMSCs. Using a mixed lymphocyte reaction (MLR) assay, we also compared the ability of the different donor BMSCs to suppress T-cell proliferation, another measure of their immune-suppressive ability. We found that the BMSCs from a donor that induced the most IL-10 production were also the most efficient in suppressing T-cell proliferation. Transcriptome studies showed that the most potent BMSC batch also had higher expression of several known key immunomodulatory molecules such as hepatocyte growth factor (HGF), PDL1, and numerous members of the PGE2 pathway, including PTGS1 and TLR4. Multiplex ELISA experiments revealed higher expression of HGF and IL6 by the most potent BMSC donor. Based on these findings, we propose that THP-1 cells may be used to assess BMSC immunosuppressive activity as a product characterization assay.

## 1. Introduction

BMSCs (frequently referred to as mesenchymal stromal/stem cells or MSCs) are a key component of the bone marrow. They create and maintain a three-dimensional microenvironment and provide important factors that support hematopoiesis [1,2]. The immunomodulatory potential of cultured BMSCs has been established for close to two decades [3,4,5,6]. There is a plethora of in vitro, preclinical, and clinical studies that identify potential clinical settings where BMSCs may be therapeutic, and there are ongoing clinical trials for a variety of these diseases [7]. Many of these studies have also shed light on important mechanisms of BMSC-mediated immunosuppression, although the overall picture is not yet clear [8,9]. While the number of registered BMSC trials is steadily increasing, and several in vitro potency assays (assays that measure the ability of BMSCs to suppress various immune functions, mostly T-lymphocyte proliferation) have been developed, there is still no consensus on how to predict clinical efficacy of various BMSC products. Co-culture systems allow us to study the interactions between samples of candidate BMSC batches and the immune cells that drive the in vivo immunosuppressive changes following BMSC administration. Although BMSCs can suppress various immune cell types, their interactions with T lymphocytes [10,11,12] and monocyte/macrophages [13,14] have attracted the most attention over recent years. Interestingly, there are several reports that suggest that the suppression of T-lymphocyte functions is macrophage-dependent [15,16]. Moreover, in certain disease settings, such as in sepsis and sarcoidosis, we have also shown that the primary target of BMSC-driven immunomodulation is the monocyte/macrophage system [17,18]. Given that most potency assays focus on investigating BMSC-T-lymphocyte interactions [10], in this study, we attempted to develop a human monocyte/macrophage-based in vitro assay utilizing the monocytic leukemia cell line THP-1 [19] to predict the immunosuppressive potential of BMSCs derived and expanded from different donors.

## 2. Results

### 2.1. BMSCs Increase IL-10 Output from THP-1 Cells

**Figure 1:**
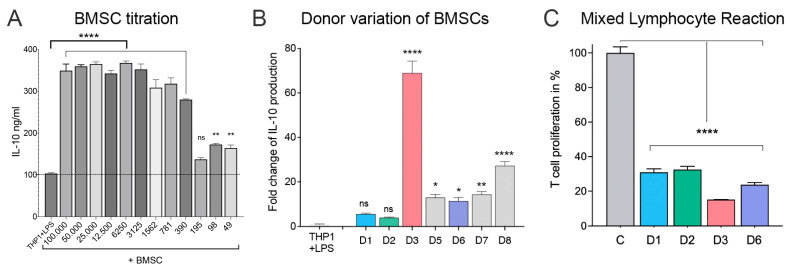
Effect of increasing number of BMSCs on THP-1-derived IL-10 production; BMSCs from donor D2 were used in the study (**A**). Effect of BMSC donor variation on IL-10 release (**B**). Suppression of T-cell proliferation by various BMSC donors (**C**). The donor cells were studied in 2–4 separate experiments. A representative experiment is shown in each graph. Statistical significance was calculated using one-way ANOVA with multiple comparisons using GraphPad Prism 9. Significance is shown by stars: ns—no significance, * *p* < 0.05, ** *p* < 0.01, and **** *p* < 0.0001.

Earlier, our laboratory has shown in mice that in LPS-stimulated co-cultures of BMSCs and macrophages, the macrophages reduce pro-inflammatory TNF-α production and increase anti-inflammatory IL-10 secretion [17]. Here, we show that, like in mice, the presence of human BMSCs in an LPS-stimulated co-culture setting also leads to increased IL-10 production by THP-1 cells (Figure 1A). To find the optimal ratio between the two cell types, the number of THP-1 cells was kept standard at 100,000 cells/per well, while the number of cells from BMSC donor D2 was reduced by 2-fold serial dilutions starting from 100,000 down to 49 cells per well. The majority of BMSC concentrations resulted in a significant increase in THP-1 IL-10 output compared to LPS-stimulated THP-1 cells not in co-culture with BMSCs. BMSC concentrations over 3125 cells per well caused a relatively consistent ~3.5-fold increase in IL-10 production, while concentrations below 390 BMSCs per well did not show a significant increase in IL-10 production (Figure 1A). Interestingly, although to a much smaller extent, even 49 BMSCs seem to have a statistically significant (*p* < 0.01) effect on the THP-1-derived IL-10 production when placed in culture with 100,000 THP-1 cells, which is a ratio of ~1 BMSC to 2000 THP-1 cells.

Next, we compared the effect of BMSCs from various donors (D1, D2, D3, D5, D6, D7, D8) on the same batch of THP-1 cells. Out of the seven examined BMSCs samples, five caused a ~3–10-fold increase in IL-10 production, one donor (D8) produced a ~30-fold increase, while another donor’s BMSCs (D3) resulted in a close to 70-fold increase in THP-1-derived IL-10 secretion compared with IL-10 derived from LPS-stimulated THP-1 cells that were not in co-culture with BMSCs. Of the seven donors studied, donor D3 stood out to be the most immunosuppressive, generating the highest IL-10 output from THP-1 cells (Figure 1B).

### 2.2. MLR

While the IL-10 measurements are suggestive of the ability of donor BMSCs to switch macrophages to an anti-inflammatory state (increased IL-10 production), the MLR is indicative of their efficiency in suppressing T-cell proliferation [20]. Using the four available BMSC donors from our earlier studies (D1, D2, D3, and D6) in the same passage that we used for the IL-10 studies, we wanted to see how efficiently they suppress the proliferation of T cells. Using two different batches of responder cells, we found that all BMSCs were successful in suppressing T-cell proliferation. From the four donors examined, the most efficient, D3, suppressed T-lymphocyte proliferation by 83.7 ± 1.8% (Figure 1C).

### 2.3. Gene Expression Analysis and Secretion Profile of Various BMSC Batches

We next examined the gene expression profile of four BMSC donors, including D3, the strongest IL-10 inducer.

**Figure 2:**
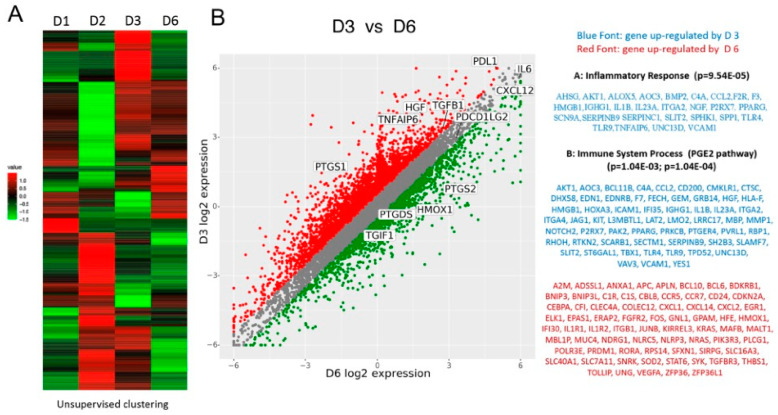
Microarray heat maps of differentially expressed mRNAs. The differentially expressed genes are shown by BMSCs from different donors. Heat maps of differentially expressed genes were generated using an unsupervised clustering method, in which the columns represent BMSCs from 4 donors (D1, D2, D3, D6), while the rows stand for the genes. Upregulated genes are represented in red, and downregulated genes are represented in green. (**A**). List of select, differentially expressed genes in immunomodulatory and anti-inflammatory pathways are listed. The genes expressed by BMSCs from two donors (D6 and D3) are plotted on the *x*-axis and *y*-axis, respectively, with the interested genes highlighted on the plot. The genes that are involved in the inflammatory response and immune system process pathways were provided and highlighted in accordance with their expression levels (**B**).

Hierarchical clustering based on differentially expressed genes revealed a clear separation between the highly immunosuppressive D3 and the other samples (Figure 2A). An in-depth comparison of D3 and D6 (the best and a medium performer) was conducted. We identified several genes differentially expressed within 2.0 FC, *p* < 0.05; 640 genes were upregulated in D3, and 755 genes were upregulated in D6. A unique list of genes upregulated in D3 but not in D6 was created. Several molecules known to play an important role in the immunosuppressive function of BMSCs were uniquely upregulated in D3. These included COX1, a key enzyme in prostaglandin synthesis; HGF, a secreted growth factor with potent immunomodulatory properties; and PDL-1. When comparing D3 to D1 and D2 and D6, TSAIP6 (TSG-6), a multifunctional protein with anti-inflammatory and tissue-protective roles was also upregulated. Also, COX2 and HMOX1 were downregulated in all donors compared to D3. In addition, the LPS receptor TLR4, previously shown to amplify the immunosuppressive properties of BMSCs, was also highly expressed in D3 but not in D6-derived BMSCs. Other D3 dominant molecules with important reparative functions included VCAM-1, EDNRA, and BMPER. VCAM-1 supports angiogenesis, EDNRA mediates induction of CDH2 and VEGF by EDN, thereby contributing to tissue restoration after myocardial infarction, while BMPER is known to play an important role in osteogenesis (Figure 2B).

Subsequently, multiplex ELISA was performed to compare the secretion of selected immune-modulatory cytokines and growth factors among BMSC donors. In concert with our microarray data, D3-derived BMSCs secreted more than three times the amount of HGF compared to D6 and other BMSC donors. IL-6, a pleiotropic cytokine best described as an immunomodulatory rather than immune-suppressive cytokine [21], was also more abundant in D3-derived BMSCs. IL10 secretion was minimal in all donor BMSCs. The expression of TGF-β1 and TGF-β2 appeared somewhat lower in D3-derived BMSCs compared with others, while SDF1b was reduced more than 6-fold compared with the D6 batch. No difference was seen in VEGF and PDGF-BB secretion (Table 1).

## 3. Discussion

An in vitro potency assay to predict the in vivo efficacy of BMSCs and to pinpoint the mechanism of action of the BMSC products has been long sought after, and several intriguing studies have been published on this topic. An ideal in vitro screening assay should predict in vivo interactions between BMSCs and key cell types that are able to create and maintain an anti-inflammatory milieu. Following the first publication that reported infusion of BMSCs to treat acute graft-versus-host disease (GVHD) [22], a primarily T-cell-driven disease, most laboratories focused their efforts on exploring interactions between stromal cells and T lymphocytes. BMSCs were shown to suppress various subpopulations of CD4^+^ helper T cells (Th1, Th2, Th17) as well as CD8^+^ cytotoxic T cells while augmenting the generation of regulatory T cells [11,12].

Several lymphocyte-based potency assays have been developed over the past few years. Bloom et al. suggested a flow cytometry-based CD4 lymphocyte proliferation assay, where suppression values are determined as the proliferation ratio of BMSC-exposed and BMSC-naïve lymphocytes [23]. Some studies aimed to standardize BMSC potency assays using a single donor-derived BMSC cell line or immortalized suppressor cells such as the Karpas 299 line (a human non-Hodgkin’s large cell lymphoma cell line) [24]. Pooled PBMCs instead of individual batches were also shown to significantly reduce inter-assay variability [25,26].

In a recent paper, Boberg et al. studied 11 patients who were receiving repeated infusions of BMSCs to treat chronic steroid-refractory GVHD, and they analyzed the response to therapy of all patients. They found distinct differences between responders and “non” responders and suggested CXCL9 and CXCL10 as early biomarkers for responsiveness to BMSC treatment. They also called attention to a very important point that not only the BMSC donors need to be studied, but the immune phenotype of the recipient must also be considered [10].

Following the 2016 consensus statement on BMSC potency assays by the International Society for Cell Therapy (ISCT), several elegant studies have surfaced utilizing assay matrix approaches to decipher complex BMSC immune cell interactions and predict immunosuppressive potency of BMSC cell products [27]. Utilizing a STAT phosphorylation assay matrix, Chinnadurai et al. reported that STAT1 and 3 phosphorylation in BMSCs following exposure to BMSC/PBMC conditioned medium predicts T-cell suppressive ability of BMSCs [28]. A similar assay matrix approach was employed in two studies, which demonstrated that the degree of BMSC-driven T and B cell suppressive capacity correlates either directly or inversely with baseline and inducible BMSC chemokine and cytokine secretome signatures [29,30]. Interestingly, these secretion profiles may only be bystander predictors of suppression potency with no inherent effect on BMSC-mediated antiproliferative action on T cells.

When exposing peripheral blood mononuclear cells (PBMCs, which represent a combination of monocytes and lymphocytes) to BMSCs, the latter suppresses lymphocyte proliferation in a monocyte-dependent manner [4]. Several other groups, including ours, have provided additional evidence that monocytes and macrophages play a critical role in BMSC-induced immunomodulation both in vitro and in vivo [16,17,31]. This is achieved by the reprogramming of monocytes/macrophages from an inflammatory phenotype (often referred to as M1) to an anti-inflammatory phenotype (M2). This phenotype switch is marked by a decrease in M1 cell surface markers such as CD80 and the appearance of M2 markers like CD206 [31]. Importantly, this paradigm shift also manifests in a marked increase in IL-10 production while the production of TNF-α is suppressed. This phenomenon has been observed both in vivo in preclinical animal models as well as in vitro in BMSC/macrophage co-culture systems [17]. These are ELISA-based assays where BMSCs and either primary or immortalized macrophages are cultured together, and following LPS priming, secreted IL-10 levels are measured. In a multi-well format, this can serve as a robust assay to determine BMSC donor-dependent variations and select BMSCs with the greatest immunosuppressive potential from various donors. Although flow cytometry-based, a similar approach was taken by Ribeiro et al. [32] when whole blood monocytes were exposed to BMSCs and monocyte TNF production was quantified. In our present study, we utilized a THP-1 cell line-based co-culture system, which helped us identify a BMSC donor with the greatest immunosuppressive potential. The assay could be used as reference material for research and/or potency assay development. THP-1 cells are monocytic leukemia cells that have been used extensively to study myeloid cell functions. They recapitulate several monocyte/macrophage characteristics, such as M1/M2 polarization, as well as LPS-stimulated TNF-α and IL-10 output [33]. In all our assays, BMSCs do not make IL-10 even after LPS stimulation, or whatever they make, is at or under the detection limit of our ELISA assays. As expected, BMSCs caused a dose-dependent increase in IL-10 output from THP-1 cells. Importantly, the BMSCs that resulted in the greatest IL-10 release from THP-1 cells were also able to mitigate T-cell proliferation the most in an in vitro mixed lymphocyte proliferation assay (MLR). In our experience, other monocyte/macrophage responder cell lines, such as U937 cells, could be used instead of THP-1 cells, but the latter seems to be releasing higher amounts of IL-10 and seems more reproducible. The goal is to utilize a readily available, well-characterized responder cell line with predictable response that is easy to quantify. IL-10 is a known immunosuppressive factor with the ability to diminish T-cell functions, including cell proliferation [34]. Although it is only one of the numerous factors responsible for BMSC-mediated T-cell suppression, its relative expression may serve as a predictor of BMSC potency; therefore, we feel that monocyte/macrophage-based potency assays may have an important role in predicting in vivo efficacy of BMSCs. Nevertheless, before THP-1-based assays can be recommended as a universal screening tool, larger comparative studies are needed utilizing more BMSC donors, along with more correlation of in vitro results with clinical outcomes.

## 4. Materials and Methods

### 4.1. Cells

Primary human BMSCs from iliac crest biopsies (donors D1, D2, D3, D5, D6, D7, D8) were isolated, characterized, cultured, stored, and reconstituted as described earlier [20] under protocol 10-CC-0053 (NCT01071577). Deidentified patient characteristics can be found in Table 2.

THP-1 monocytes were obtained from ATCC (American Type Culture Collection) and cultured in RPMI-1640 (Gibco), supplemented with 10% FBS 1% PenStrep in 96-well plates at a density of 1 × 10^5^ cells/well.

### 4.2. Co-Culture of BMSCs with THP-1 Cells

In the donor comparison experiments, BMSCs were co-cultured overnight with THP-1 cells in a ratio of 1:10, respectively, followed by stimulation with 1 µg/mL bacterial lipopolysaccharide (LPS, Sigma Aldrich, St. Louis, MO, USA) for 24 h. Supernatants were then assayed for IL-10 by ELISA (R&D Systems. DY217B, Minneapolis, MN, USA).

In the titration experiments, BMSC cells in decreasing concentrations were added to THP-1 cells (ATCC, TIB-202), starting from a 1:1 ratio to a final dilution of 1:2028. Each condition was assayed in technical triplicates, and the co-culture experiments were repeated 2–4 times. Comparison of BMSCs from 7 donors (D1, D2, D3, D5, D6, D7, D8) were performed using the THP-1 co-culture method.

### 4.3. MLR

The immunosuppressive properties of BMSCs from different donors were compared using the MLR assay performed at SAIC—Frederick MD/National Cancer Institute. This assay is used to test the ability of the BMSCs to affect T-cell proliferation and measure the immunosuppressive properties. Two different batches of responder cells (T cells) were co-cultured with 2500cGy irradiated stimulator peripheral blood mononuclear cells (PBMCs) at a concentration of 1 × 10^5^ cells/well. BMSCs from different donors were added at a 1 × 10^4^ cells/well concentration (1:10 BMSC/responder ratio), and the cells were incubated for 6 days. On the day of harvest, 0.5μCi of 3H-thymidine (3H-TdR) was added to each well for 4 h, with lymphocyte proliferation measured using a liquid scintillation counter. The effect of BMSCs on the proliferation of lymphocytes was calculated as the percentage of the proliferative response of the positive control without the presence of BMSCs, where the positive control was set to 100%. The experiments were performed three times for each variable described. For more details, see [20]. BMSCs from 4 donors (D1, D2, D3, D6) were used for the MLR.

### 4.4. Microarray Gene Expression Analysis

Total RNA was extracted using miRNeasy Mini Kit (Qiagen, Hilden, Germany) and assessed using Nano-Drop 2000 (Thermo Scientific, Wilmington, DE, USA). Microarray expression experiments were performed on 4 × 44 K Whole Human Genome Microarray (Agilent Technologies, Santa Clara, CA, USA). Images of the arrays were acquired with a microarray scanner G2505B (Agilent Technologies), and image analysis was performed using feature extraction software version 9.5 (Agilent Technologies). The data (accession number GSE34303) were submitted to Gene Expression Omnibus (GEO). For more details, see [20].

### 4.5. Cytokine and Growth Factor Analysis

BMSC culture supernatants were evaluated for concentrations of known growth factors and cytokines using SearchLight Protein Array Analysis (Aushon BioSystems, Billerica, MA, USA). Cell debris was removed by centrifugation for 10 min (1400 rpm) from the culture supernatant, which was then stored at −80 °C. The supernatants of BMSCs were evaluated for human fibroblast growth factor (hFGF), human hepatic growth factor (hHGF), human Interleukin-10 (hIL-10), human Interleukin 6 (hIL-6), human transforming growth factor beta 1 (hTGF-beta1), human transforming growth factor beta2 (hTGF-beta2), human stromal-derived factor (hSDF-1beta), human platelet-derived growth factor BB (hPDGF-BB), and human vascular endothelial growth factor (hVEGF).

## Figures and Tables

**Table 1:** Concentrations of select growth factors/cytokines in BMSC supernatants (pg/mL).

	BMSC-M *	D3	D5	D6	D7	D8
hFGFb	-	0.46	-		-	-
hHGF	-	3467	1079	1073	507	566
hIL10	-	0.35	0.46	0.25	0.24	0.28
hIL6	0.1	10,066	7452	6792	3501	4928
hPDGFb	-	0.75	1.45	1.07	1.2	0.66
hCXCL12b	4.45	761	9629	4878	6021	5704
hTGFB1	8774	12,625	19,206	17,554	17,811	17,238
hTGFB2	2357	2345	6335	4582	4032	3860
hVEGF	1.25	2805	3648	2737	2696	1759

* BMSC-M—medium. Culture supernatants of a variety of healthy donor BMSCs were evaluated for concentrations of known growth factors and cytokines they secrete using a multiplex ELISA (SearchLight Protein Array Analysis). Red and blue fonts point at factors that are significantly upregulated (red) or downregulated (blue) by D3, which turned out to perform best in the other assays as well.

**Table 2:** BMSC donor characteristics.

Donor	Sex	Age at Donation	Race
D1	Male	31	African American
D2	Male	30	Caucasian
D3	Female	22	Caucasian
D5	Male	22	Caucasian
D6	Female	23	Caucasian
D7	Male	27	African American
D8	Male	42	Caucasian

## Data Availability

Not applicable.
